# Unlocking Patient Resistance to AI in Healthcare: A Psychological Exploration

**DOI:** 10.3390/ejihpe15010006

**Published:** 2025-01-08

**Authors:** Abu Elnasr E. Sobaih, Asma Chaibi, Riadh Brini, Tamer Mohamed Abdelghani Ibrahim

**Affiliations:** 1 Management Department, College of Business Administration, King Faisal University, Al-Ahsaa 31982, Saudi Arabia; 2 Management Department, Mediterranean School of Business (MSB), South Mediterranean University, Tunis 1053, Tunisia; asma.chaibi@msb.tn; 3 Department of Business Administration, College of Business Administration, Majmaah University, Al Majma’ah 11952, Saudi Arabia; 4 Social Studies Department, Faculty of Arts, King Faisal University, Al-Ahsa 31982, Saudi Arabia; tabdelghani@kfu.edu.sa

**Keywords:** artificial intelligence (AI), AI in healthcare, patient resistance, perceived technological dependence, psychological variables, skepticism toward AI

## Abstract

Artificial intelligence (AI) has transformed healthcare, yet patients’ acceptance of AI-driven medical services remains constrained. Despite its significant potential, patients exhibit reluctance towards this technology. A notable lack of comprehensive research exists that examines the variables driving patients’ resistance to AI. This study explores the variables influencing patients’ resistance to adopt AI technology in healthcare by applying an extended Ram and Sheth Model. More specifically, this research examines the roles of the need for personal contact (NPC), perceived technological dependence (PTD), and general skepticism toward AI (GSAI) in shaping patient resistance to AI integration. For this reason, a sequential mixed-method approach was employed, beginning with semi-structured interviews to identify adaptable factors in healthcare. It then followed with a survey to validate the qualitative findings through Structural Equation Modeling (SEM) via AMOS (version 24). The findings confirm that NPC, PTD, and GSAI significantly contribute to patient resistance to AI in healthcare. Precisely, patients who prefer personal interaction, feel dependent on AI, or are skeptical of AI’s promises are more likely to resist its adoption. The findings highlight the psychological factors driving patient reluctance toward AI in healthcare, offering valuable insights for healthcare administrators. Strategies to balance AI’s efficiency with human interaction, mitigate technological dependence, and foster trust are recommended for successful implementation of AI. This research adds to the theoretical understanding of Innovation Resistance Theory, providing both conceptual insights and practical implications for the effective incorporation of AI in healthcare.

## 1. Introduction

Over the past few decades, digital transformation has significantly advanced in various sectors, including healthcare. Bouarar et al. (2023) emphasize that this shift towards digital healthcare is vital, as it offers doctors a comprehensive understanding of their patients’ health and allows patients to receive faster services. This transformation is driven by technological innovations, e.g., augmented reality, robotics, big data, and notably, artificial intelligence (AI). The COVID-19 pandemic has accelerated the implementation of electronic solutions by health organizations worldwide, particularly AI, enhancing patient care (Köse, 2023; Olawade et al., 2023).

AI in healthcare (AIH) is an evolving technology that allows healthcare providers to manage data by mimicking human cognitive roles with enhanced efficiency and throughput. This innovation is driving a significant change in healthcare, facilitated by the growing accessibility of healthcare data and advancements in analytical methods (Laï et al., 2020). AI’s rapid expansion in the healthcare sector is reflected in its projected market size of USD 45.2 billion by 2026 (Kishor & Chakraborty, 2022). AI in healthcare refers to analytical software capable of automating routine tasks traditionally performed by humans. Such software not only simulates human activities, but also performs complex analytical functions that were previously exclusively human tasks (Hameed et al., 2023).

AI includes a broad spectrum of technologies and methods that enable machines to accomplish jobs demanding human intelligence, e.g., visual and speech recognition, reasoning, and problem-solving (Reddy et al., 2019; J. He et al., 2019). AI in healthcare showed significant potential in areas such as clinical decision support, risk prediction, reduction of medical errors, healthcare intervention, and productivity enhancement (Reddy et al., 2019; J. He et al., 2019). It enriches the accuracy and rapidity of image review in radiology and pathology, thereby advancing medical diagnostics. Additionally, AI significantly influences patient care via virtual assistance, which affects patient–doctor interactions (Maleki Varnosfaderani & Forouzanfar, 2024). The incorporation of AI in healthcare is a key component of the broader digital transformation that is reshaping numerous industries globally (Agarwal et al., 2020).

Studies demonstrate that AI has the potential to exceed individual’s capabilities in certain areas, e.g., more accurately analyzing chest X-ray images compared to radiologists. By reducing human errors, AI allows clinicians to focus on more issues that are complex; thus, improving patient care (Yu et al., 2018). For instance, AI-assisted diagnoses in medical imaging can significantly enhance workflow efficiency by processing millions of images daily (Beam & Kohane, 2018; Chew & Achananuparp, 2022). AI chatbots provide mental health counseling, alleviating the burden on clinicians. Additionally, AI-enabled application tools allow patients to self-monitor and diagnose conditions like atrial fibrillation, skin lesions, and retinal diseases (Maleki Varnosfaderani & Forouzanfar, 2024).

Despite AI’s potential benefits in healthcare, many organizations face significant hurdles in implementing this technology (Neumann et al., 2024). These challenges include organizational, financial, technological, and human factors that complicate seamless AI adoption. Ethical concerns also arise, such as possible biases in algorithms and the risk of job loss (Sobaih, 2024). Although the AI market is growing due to its advantages, these benefits cannot be fully realized without effective implementation (Merhi, 2023). For successful AI integration, examining users’ attitudes and perceptions is essential (Sobaih et al., 2024). Capitalizing in AI technology without considering user views and acceptance may lead to wasted resources and disengaged patients, particularly in healthcare where patient engagement is critical for quality. If patients do not find AI devices useful, they may prefer physician interactions, leading to underutilization of AI tools. Therefore, identifying the variables that drive or hinder AI use in healthcare is essential to integrate these technologies (Esmaeilzadeh, 2020). Healthcare professionals also express ongoing concerns about implementing AI tools; hence, researchers need to address these concerns and design effective AI-enabled tools (Turja et al., 2020). It is crucial to link these insights to understanding user’s behavior and resistance. User’s resistance, a crucial aspect of consumer behavior, significantly influences the adoption of innovations (Seth et al., 2020; Xue et al., 2024). This resistance, often manifested as unwillingness to try new technologies, is a major determinant of an innovation’s success or failure, and presents a critical challenge for organizations implementing new technologies (Heidenreich & Kraemer, 2016; Talwar et al., 2020). Health organizations must understand the motives of consumer resistance to mitigate AI implementation failures and develop strategies to enhance its usage (Ram, 1987; Xue et al., 2024). Understanding the roots of resistance and non-adoption is essential for effective innovation management (Joachim et al., 2018).

From a theoretical standpoint, the current literature is deficient in different aspects. First, studies focused on the acceptance and resistance of different technologies like the Internet of Things (IoT) (Hajiheydari et al., 2021; Ju & Lee, 2021), blockchain (Dwivedi et al., 2023; Ameyaw et al., 2023), M-payment (Behera et al., 2023), wearable devices (Tsai et al., 2020), FinTech (Irimia-Diéguez et al., 2023), and chatbots (Ayanwale & Ndlovu, 2024). However, little research has been directed towards AI technology adoption from the user’s perspective (Chew & Achananuparp, 2022). Second, earlier research has considered AI in numerous industries. This includes factors affecting AI in the food supply chain (Dora et al., 2022), education (Hasanein & Sobaih, 2023; Sobaih et al., 2024), research and publication (Sobaih, 2024), accounting for SMEs (Rawashdeh et al., 2023), hospitality management (Rasheed et al., 2023), recruitment (J.-Y. Kim & Heo, 2022), and public administration (Madan & Ashok, 2023). However, limited research adopted a grounded theory approach to evaluate the motives and challenges to the effective deployment of AI in healthcare (W. He et al., 2021; Singh et al., 2024). Third, existing studies on technology acceptance in healthcare have primarily focused on design and implementation from the service provider’s perspective, neglecting patients’ perceptions and behavioral aspects related to technology usage (Holden & Karsh, 2010). Fourth, early studies on technology adoption emphasized reasons for adoption, but often ignored factors hindering adoption (Chew & Achananuparp, 2022; Yang et al., 2024). They relied on models, e.g., “Technology Acceptance Model” (TAM) (F. D. Davis et al., 1989), “Unified Theory of Acceptance and Use of Technology” (UTAUT) (Venkatesh et al., 2003), and its updated version, the UTAUT 2 (Venkatesh et al., 2012). In addition, Expectation Confirmation Theory (Talwar et al., 2020) and Diffusion of Innovation Theory (Kaur et al., 2020) focused more on initial acceptance than long-term use and barriers to adoption (F. D. Davis et al., 1989; Venkatesh et al., 2003, 2012; Talwar et al., 2020; Kaur et al., 2020).

However, these models exhibit some limitations. First, they are more suitable for assessing users’ initial inclinations towards adopting a newly introduced technology (F. D. Davis et al., 1989; Venkatesh et al., 2003, 2012) rather than delving into users’ long-term usage behaviors. Second, such models tend to concentrate on positive factors contributing to technology acceptance (Choudrie et al., 2018; Khanra et al., 2020) rather than addressing obstacles that impede or delay its adoption. Modern scholarship recognizes that motivators for adoption do not fully explain resistance (Claudy et al., 2015), emphasizing the necessity to understand the complexities of consumers’ reluctance to embrace new technologies (Nel & Boshoff, 2019; Yang et al., 2024). Previous studies have primarily explored challenges associated with AI technology adoption qualitatively, without incorporating relevant quantitative analysis (Dhagarra et al., 2020; Pai & Huang, 2011). Previous research has identified key factors influencing resistance to healthcare information technology, considering it as a broad concept (Bhattacherjee & Hikmet, 2007; Greenhalgh et al., 2014; Nilsen et al., 2016; Mani & Chouk, 2018). These factors include technology-specific aspects like usability challenges (Laukkanen, 2016), organizational dynamics (Lebcir et al., 2021), diffusion issues (Al-Dhaen et al., 2023), and trust concerns (Asan et al., 2020), with limited attention given to psychological factors. Moreover, earlier research on resistance to innovative technologies have mainly relied on Ram and Sheth’s model (1989), which has been applied across various contexts such as online and mobile banking (Laukkanen, 2016) and smart products (Mani & Chouk, 2018). Nonetheless, such a model has drawbacks. Firstly, it is based on outdated concepts that may not fully fit the digital era (Heinze et al., 2017). Secondly, its psychological barriers encompass only image and tradition, overlooking technological vulnerability and ideological barriers. In light of these considerations, this research identifies the psychological variables that influence patient resistance to AI use in healthcare. This study extends Ram and Sheth’s model (1989) to delve into the influences of the need for personal contact, perceived technological dependence, and general skepticism toward AI.

This paper adds to the scholarly discourse on AI adoption in healthcare by addressing several gaps in the existing literature. Firstly, it narrows its focus to the specific domain of AI within healthcare, an area that has received comparatively less attention than other technological advancements. Secondly, this study delves into the perceptions and behaviors of patients, who represent the ultimate end-users in healthcare technology adoption, thereby offering valuable insights into user-centric considerations. Notably, this research distinguishes itself by centering on patients’ resistance towards AI in healthcare, with a meticulous examination of psychological determinants such as the need for personal contact, perceived technological dependence, and general skepticism, utilizing an extended model derived from Ram and Sheth (1989). Moreover, the research approach undertaken is rigorous and comprehensive. It employs a sequential mixed-method approach, integrating qualitative analysis to uncover nuanced insights and quantitative data to validate findings. This methodological rigor ensures a multifaceted understanding of the complexities surrounding patient attitudes and behaviors toward AI adoption in healthcare. Consequently, this research contributes unique and sophisticated insights that not only address patient concerns, but also provide actionable recommendations to enable effective incorporation of AI technologies in healthcare settings.

The following section includes a comprehensive literature review, presenting background insights into the application of AI in healthcare, along with relevant studies addressing resistance to AI in this field, and proposes a conceptual model and research hypotheses based on two phases of study. The third section elaborates on the research design for the two phases of this study, which started with a qualitative study and then a quantitative study. The fourth section reports the findings, followed by a discussion. This paper ends with its limitations and directions for further studies.

## 2. Literature Review

AI, a highly promising area of research, spans various industries, including healthcare, where it is reshaping organizational methods and practices (Olawade et al., 2023; Yu et al., 2018). AI models mimic human intelligence, imitating cognitive processes, e.g., problem solving and learning (Russell & Norvig, 2016; Shankar, 2018; UKRI, 2022). AI is designed to do jobs usually done by humans by utilizing technologies like machine learning and natural language handling (Dwivedi et al., 2019; Kaplan & Haenlein, 2019; Choudhary et al., 2022). Hameed et al. (2023) highlight its role in revolutionizing patient care and industry paradigms. This section delves into AI’s transformative impact on healthcare, specifically, its applications in clinical settings, and an examination of resistance models and AI resistance studies in healthcare.

### 2.1. AI Applications in Healthcare

Recent advancements have spurred the development of numerous AI applications in healthcare, including diagnostic tools, predictive analytics, and personalized medicine (Bajwa et al., 2021; Johnson et al., 2020). The surge in AI applications is attributed to improvements in techniques such as deep learning and the availability of extensive data. AI algorithms are generally classified under machine learning (ML), with deep learning (DL) as a subset of ML, and natural language processing (NLP) as a specific application area of AI, each with distinct strengths and applications (Maleki Varnosfaderani & Forouzanfar, 2024). Machine learning algorithms assist in making decisions without explicit programming, playing a pivotal role in healthcare. Supervised learning is used to develop predictive models based on trained data, while unsupervised learning identifies patterns or clusters, such as novel disease subtypes (Eloranta & Boman, 2022). Reinforcement learning, which involves learning through trial and error, shows promise for personalized treatment optimization (Coronato et al., 2020).

Deep learning, a subdivision of ML, uses neural networks with multiple layers to explore complex data. A significant achievement in this field is the development of deep learning algorithms for medicinal image analysis, leading to highly accurate diagnostic systems for conditions like cancer, heart disease, and eye diseases (Davenport & Kalakota, 2019; Ahuja, 2019). For instance, Neuralink is an innovative AI technology that integrates a wireless brain chip into the human brain, enabling persons with severe paralysis to control devices, e.g., robotic limbs and smartphones, with their thoughts (Agrawal, 2024). NLP algorithms permit computers to read human language, making them valuable for extracting meaningful information from unstructured data, e.g., clinical notes. This aids diagnostics (Zhou et al., 2022). These AI applications not only enhance patient outcomes, but also reduce costs. AI integration in healthcare was found to significantly lower expenditures in comparison to traditional diagnostic approaches, saving from 3.3 and up to 15.2 h per day, and reducing costs by USD 1667 to USD 17,881 per day per hospital (Khanna et al., 2022). Table 1 shows various AI technologies utilized across different applications within healthcare.

Although AI in healthcare has undergone several developments, it faces numerous hurdles that need to be addressed for common implementation. The ethical and practical drawbacks encompass several critical issues. AI’s susceptibility to perpetuating biases emerges as a top concern, as it can lead to unfair treatment outcomes, necessitating diverse dataset training and ongoing monitoring (Fletcher et al., 2021). Privacy is another critical concern due to the vast data needs of AI systems, necessitating secure storage, anonymization, and transparent data policies (Murdoch, 2021). Moreover, informed consent poses an intricate issue, demanding clear communication on data use, risks, benefits, and human oversight in AI decisions (Iserson, 2024). Data quality is pivotal, influencing AI’s efficacy in drug discovery, and underscoring the need for improved data standards (Olawade et al., 2023). Additionally, AI models’ vulnerabilities to manipulation and limited generalization highlight the need for robustness and adaptability (Jiang et al., 2017). The absence of clear regulatory frameworks further complicates matters, highlighting the need for established guidelines to navigate AI’s complex landscape in healthcare. The readiness of doctors and patients for this technology is still another significant obstacle. These actors have been slow adopters of fresh technology, preferring to use tried-and-true techniques to provide clinical treatment (Lapointe & Rivard, 2006).

Resistance and adoption concepts are complex, and their causes may differ because of the nature of service and the type of technology. This study will focus on the causes of AI resistance from the patient’s perspective. The decision to focus on the patient’s perspective in this research is driven by several important factors. Patients, being the end-users of AI technologies in healthcare, have an essential role in determining the success of these innovations. By understanding their concerns and resistance towards AI, we can identify potential obstacles and develop targeted solutions. Moreover, considering the diverse backgrounds of patients provides valuable insights into the ethical and privacy aspects of AI adoption. Engaging patients in the research process empowers them and promotes patient-centered healthcare innovation.

### 2.2. A Comprehensive Analysis of Innovation Resistance Models

Before presenting resistance models, it is necessary to create clear thoughts of consumer resistance to innovations, including digital innovations (Leong et al., 2020). This concept has been relatively underutilized, leading to its ongoing development and lack of well-defined boundaries (Migliore et al., 2022). The Innovation Resistance Theory (IRT), originally developed by Ram (1987) and subsequently redeveloped by Ram and Sheth (1989), provides valuable understandings into handler behaviors. This theory posits that resistance to innovations is a normal reaction to the changes they bring, emphasizing the importance of understanding the psychological factors involved in facilitating the acceptance and spread of innovations (Talwar et al., 2020). In the context of information systems, user resistance is the adverse reactions or objections to perceived changes brought about by new system implementations. Such resistance typically stems from perceived threats associated with the new system and can manifest as rejection, opposition, or delay (Markus, 1983). Sadiq et al. (2021) highlights that consumer resistance to innovation results from a rational assessment of innovations that might disrupt established norms and conflict with existing worldviews. Consumer resistance, therefore, can be understood as the reluctance to adopt changes due to contentment with the current state or conflicts affecting their belief systems (Ram & Sheth, 1989). In the situation of patient resistance to AI in healthcare, this resistance represents barriers to adopting AI technologies influenced by personal, situational, contextual, regulatory, and product-related factors. These factors include age, innovation, preference for the status quo, cultural aspects, government regulations, and product characteristics. Hence, this research defines patient resistance as hurdles to the usage of any innovation resulting from AI technology (Ram & Sheth, 1989). Several theories have been proposed to recognize the phenomenon of resistance to innovation and the variables that affect individuals’ reluctance to adopt new systems. Table 2 is a summary of some of the prominent resistance theories along with their key concepts and authors.

### 2.3. Prior Studies on AI Resistance

In the realm of AI resistance research, Alsheibani et al. (2019) examined AI adoption barriers in Australian organizations through the TOE framework and an online questionnaire, providing valuable insights and a research agenda for executives and managers. M. K. Lee and Rich (2021) focused on human perceptions of AI in healthcare, emphasizing the influence of group-based medical mistrust and social group differences. Strohm et al. (2020) identified several key barriers to implementing AI applications in radiology, including unreliable performance, unstructured execution processes, uncertain added value for clinical practice, and varying levels of acceptance and trust among adopters. Cadario et al. (2021) identified resistance to medical AI, attributing it to challenges in consideration of algorithms and misleading consideration of human medical decision-making, proposing interventions to promote AI adoption. Gao et al. (2020) analyzed social media data, revealing positive attitudes toward AI doctors, tempered by concerns about technology maturity and company trustworthiness. Bhattacherjee and Hikmet (2007) offered a model of physician resistance to HIT usage, highlighting perceived threat and compatibility as key factors in resistance intentions. Gaczek et al. (2023) explored consumer resistance to AI healthcare recommendations, discovering the impact of diagnosis trustworthiness and health anxiety, with social proof as a mitigating factor. Mugabe (2021) noted that poor experience hinders the use of AI in radiation oncology. Jussupow et al. (2022) investigated professional identity threats in medical professionals’ AI resistance, examining perceived self-threat, temporal distance of AI, and differences between medical students and professionals. Chaibi and Zaiem (2022) found that hurdles to AI adoption among physicians include a poor infrastructure, including financial resources, specialized training, performance risks, perceived costs, technology dependency, and fears of AI replacing human jobs. Table 3 summarizes the studies on AI resistance.

In this context, our study differs by focusing on patients’ resistance to AI in healthcare, with emphasis on psychological variables, e.g., the need for personal contact, perceived technological dependence, and general skepticism. We employ a sequential mixed-method approach, combining qualitative analysis for deeper insights and quantitative data for validation, contributing unique insights to address patient concerns and enhance AI adoption in healthcare settings. While previous studies have made valuable contributions to understanding resistance factors in various contexts, there is a notable research gap concerning patients’ resistance to AI adoption in healthcare.

## 3. Research Design

Given the multitude of factors exerting influence on patient resistance to AI, we employed sequential mixed-methods including two phases. The qualitative data collection precedes the quantitative phase. This qualitative study was undertaken to discern the most adaptable determinants that shape AI resistance within the Tunisian context.

### 3.1. The Conduct of the Qualitative Study

Our qualitative phase was undertaken with a sample of patients using an interview guide that aims to identify the most critical factors affecting AI acceptance in the Tunisian healthcare industry. These interviews informed the development of a survey, which was distributed online to a wider audience. Through this approach, we aimed to gather comprehensive data on the variables affecting patient use of AI.

#### 3.1.1. Interview Guideline

**Figure 1:**
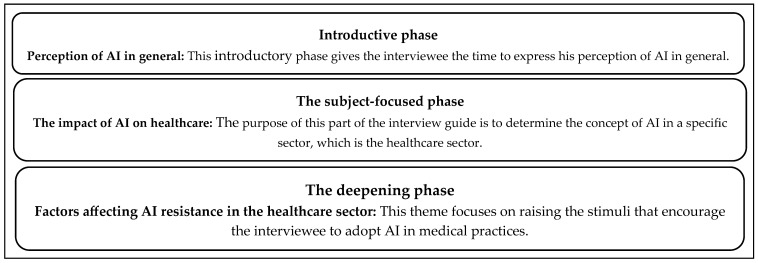
Interview guideline adopted in this study.

The interview guide represents both a guideline for conducting the interview and a point of reference that reminds us of the fundamental themes to be dealt with during the face-to-face meeting. This interview guide is structured around themes from the literature, the research problem, and a little less the researcher’s intuition (Charfi, 2012). The interview guide was organized into three phases, namely, an introductory phase, a subject-focused phase, and a phase of deepening based on the structure presented by Evrard et al. (2009) (Figure 1).

#### 3.1.2. Data Collection of the Qualitative Study

Individual interviews were conducted during the winter of 2022/2023. Most were face-to-face, but Microsoft Teams was used when necessary. This research focused on AI in healthcare, with confidentiality and anonymity assured. All interviews were audio-recorded on the condition of consent in order to ensure that such data were accurately analyzed. The interview length was between 30 and 45 min. Verbatim transcription was employed to preserve the authenticity and nuance of participants’ responses, ensuring accuracy and credibility in the analysis (Rowlands, 2021).

#### 3.1.3. Participants

Different non-probability sampling was adopted, particularly, purposive and snowball sampling, as well as convenience sampling for easily accessible and motivated participants. We undertook semi-structured meetings with a diverse sample of 43 individuals, encompassing various genders, ages, and occupations. Out of the 43 participants, the majority (73.2%) were female. The age was from 21 to 60 years old, with the largest age group being 36 years old, representing 10.5% of the sample. In terms of occupations, the sample includes workers (7%), IT engineers (14%), professors (28%), students (19.5%), teachers (2.3%), unemployed individuals (4.7%), managers (2.3%), senior directors (2.3%), those in liberal professions (2.3%), and retirees (4.7%). The sample exhibits a diverse representation of individuals from various genders, age groups, and professional fields.

#### 3.1.4. Data Analysis for the Qualitative Study

Thematic analysis was utilized to thoroughly examine the data from interviews and uncover common patterns and trends. This rigorous process involved analyzing the data using QSR NVivo (version 12) software, where interviews were systematically coded and categorized into meaningful themes and subthemes.

The unit of analysis was the theme, defined as “a statement about a topic. That is, a sentence, or a compound sentence, usually a summary or a condensed sentence, under which a vast set of singular formulations can be affected” (Berelson, 1952; Bardin, 2007). After transcription, the text was divided according to main themes and sub-themes. Data categorization followed a systematic open coding process, which adheres to Bardin’s (2007) criteria:

- Homogeneity: units of analysis belong to the same register.
- Mutual exclusion: a unit can only be assigned to one category.
- Relevance: categories align with the content and theoretical framework.
- Productivity: results must be information rich.
- Objectivity: different coders should achieve the same results.

Themes were identified as a priori (based on the literature) and a posteriori (emerging during coding). Using NVivo software, themes were represented as nodes and sub-themes as sub-nodes. The results were processed to retain the most significant information while ensuring alignment with the study’s objectives (Evrard et al., 2009). The following steps were undertaken:

- Simplification of answers without losing detail.
- Identification of plausible themes, aspects, and typologies.
- Identification of variables and their interrelations.
- Development of tables highlighting results using simple statistical operations (e.g., percentage calculations).

To validate findings, the research team engaged in comprehensive discussions, and two researchers cross-checked quotes against the identified themes. The analysis outcomes were aligned with established concepts in the literature, enhancing the credibility and reliability of the findings (Charfi, 2012).

#### 3.1.5. Reliability and Validity of Qualitative Research

In order to assess the reliability of coding, Weber (1990) recommends examining three criteria. First, the stability of the coding passes by the evaluation of the results of the coding carried out repeatedly by the same encoder. Then, the precision of the coding aims at the evaluation of the coding position by referring to a standard established in advance. This criterion is very rarely applied, except in exceptional cases, especially in research that has set up standard coding. Finally, reproducibility is also called reliability inter-coders. The latter aims to make comparisons between the results of the coding of the same text carried out by various coders. Thematic analysis of the qualitative data using NVIVO software revealed one major theme, “Psychological Factors Affecting Patient Resistance to AI”, consisting of three sub-themes: The need for Personal Contact, Perceived technological Dependence, and General skepticism (Table 4).

**Figure 2:**
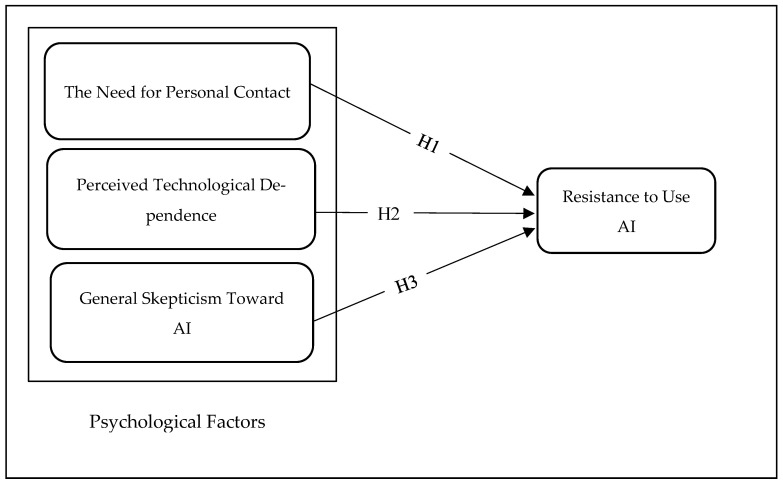
The conceptual model.

To establish our hypotheses for the conceptual model (Figure 2), we correlated qualitative findings with current studies, confirming the critical role of these factors in shaping patients’ resistance to AI. This study focuses on psychological barriers, as Ram and Sheth (1989) defined psychological factors as those that arise when innovation upsets consumers’ prior beliefs, connected with the image of innovation and tradition (Ram & Sheth, 1989). The results are in line with the categorization of resistance drivers outlined in innovation resistance theory (IRT). We justify the adoption of IRT in current research for numerous causes. First, IRT was adopted in many user innovation contexts, e.g., mobile banking, smart products, eco-friendly cosmetics, and healthcare (Laukkanen, 2016; Mani & Chouk, 2018; Sadiq et al., 2021; Migliore et al., 2022; Chen et al., 2022; D. H. Kim & Lee, 2020; Talwar et al., 2020; Hajiheydari et al., 2021). The extensive adoption of this theory reflects its validity in exploring consumer resistance toward AI technology. Second, earlier studies confirmed IRT’s effectiveness in understanding customer resistance to innovations, distinguishing it from other theoretical frameworks like UTAUT, TAM, and DOI, despite these theories not explicitly examining resistance to innovations (Leong et al., 2020; Kaur et al., 2020). The selection of IRT as our framework is rooted in its ability to elucidate consumer resistance across diverse innovation scenarios, making it the most suitable framework for our specific research objective. This aligns with Ram and Sheth’s (1989) notion that psychological barriers emerge when innovation disrupts consumers’ established beliefs, highlighting the complex interplay of psychological elements in understanding resistance to AI technology (Ram & Sheth, 1989).

### 3.2. Hypothesis Development

#### 3.2.1. The Need for Personal Contact

Based on our qualitative study, resistance to AI-based healthcare services would depend on how much individuals perceive personal contact to be necessary or desirable. Some individuals favor these services since they reduce the need for one-to-one interactions with service providers and other clients, and they find them pleasurable. On the other hand, some individuals prefer to interact with human beings rather than machines, which they view as impersonal and unable to provide personalized service. Previous studies have indicated that few individuals favor service encounters that allow for human interactions (Curran et al., 2003; Dabholkar & Bagozzi, 2002; F. D. Davis et al., 1992). Thus, it is practical to assume that those who prefer human interaction in healthcare services may be hesitant to use technologically assisted means of service providing simply because they value the human element. Therefore, we propose:

**H1.**  *Personal contact has a positive impact on patients’ resistance to use AI in healthcare.*

#### 3.2.2. Perceived Technological Dependence

The increasing use of modern ICT in many aspects of life, e.g., work, education, and social interactions, has led to a growing reliance on these technologies. According to Mani and Chouk (2017), the continuous connection of technological devices to the Internet could create both functional and psychological dependence, which may result in resistance to technology. Research has identified negative consequences of dependence, e.g., social isolation (R. A. Davis, 2001) and technostress (Shu et al., 2011), and how consumers are increasingly dependent on these technologies, which negatively affects their behavior (e.g., isolation and loss of control) (Shu et al., 2011). These results are supported by research in cyber-psychology, which highlights the adverse effects of dependence on new technologies (Young, 2004; Lu, 2008). Based on the technology use propensity index (Ratchford & Barnhart, 2012), which considers dependence as a variable that impedes the use of technology, and earlier studies (Mani & Chouk, 2017; Shu et al., 2011), we can propose that:

**H2.**  *Patients’ perceived technological dependence has a positive effect on their resistance to AI in healthcare.*

#### 3.2.3. General Skepticism

Moreover, Mani and Chouk (2018) have identified skepticism as a factor that contributes to patients’ doubts about the promises and arguments made by AI companies in their marketing. Currently, there is a widespread discourse that emphasizes the revolutionary nature of IoT devices, which are marketed as products that can transform consumers’ lives through new services. In this situation, patients may resist the adoption of AI in healthcare because of their skepticism towards the technology. This skepticism can stem from doubts about the promises and claims made by AI companies, which are often marketed as revolutionary devices that can transform healthcare. Patients may show their resistance to AI corporations perceived to be dominant market forces, and their skepticism can influence their attitudes and behaviors toward the adoption of AI. Therefore, we propose:

**H3.**  *Patients who are more skeptical of AI may be more likely to resist its use in healthcare.*

Figure 2 presents the research conceptual model.

### 3.3. The Conduct of the Quantitative Study

#### 3.3.1. Data Collection of the Quantitative Study

The data collection techniques for this research study involve the adoption of surveys to gather quantitative data on the factors influencing Tunisian patients’ resistance to AI in healthcare (Saunders et al., 2012; Creswell, 2013). Specifically, online surveys are deemed the most appropriate method for this research (Zikmund, 2010). Additionally, a combination of self-administered and interviewer-administered survey questionnaires is recommended (Saunders et al., 2012). To select suitable participants, non-probability sampling techniques, e.g., purposive sampling, convenience sampling, and snowball sampling, are employed (Cavana et al., 2001; Guarte & Barrios, 2006; Teddlie & Yu, 2007). In measuring attitudes, the Likert scale is utilized (Evrard et al., 2009). Moreover, to accommodate the primarily Tunisian sample, the questionnaire is translated into French using rigorous translation techniques (Hafeez-Baig & Gururajan, 2010). The survey was pre-tested to confirm the quality of the questionnaire, resulting in improvements in its length and clarity based on feedback from the respondents (Temessek, 2008).

#### 3.3.2. Research Methods and Instrument

To measure the construct of the need for personal contact in the context of AI resistance in healthcare, we drew inspiration from the works of Walker et al. (2002). Technological dependence variables were drawn from the Charlton (2002) scale. Moreover, skepticism towards IoT was assessed with variables developed by Morel and Pruyn (2003). Finally, the measurement of resistance to the use of AI was developed by Hseih and Lin (2017). This scale aims to assess individuals’ resistance or reluctance to use AI technology for managing their health. It consists of five measurement items that capture different dimensions of resistance. Table 5 shows the resistance factors proposed in the research model and their measurement items.

#### 3.3.3. Data Analysis of the Quantitative Study

The data analysis encompassed five distinct phases, namely, the examination of demographic information, verification of validity and reliability, exploration through exploratory factor analysis (EFA), validation via confirmatory factor analysis (CFA), and structural equation modeling (SEM). The EFA phase sought to ascertain the explained overall variance and uncover the inherent structures and dimensions of the measurement tools employed. Subsequently, the CFA phase was undertaken to examine and endorse the models utilized in this study. Lastly, SEM was used to evaluate the causal connections between the factors within the model, while employing indices to ascertain the alignment of the theoretical model with the gathered data.

#### 3.3.4. Demographics of Respondents Participating in the Quantitative Study

The gender distribution reveals that 50.7% (228) of the participants are male, while 49.3% (222) are female, indicating a nearly equivalent representation of both genders. Regarding age, the common respondents fall within the 18–24 and 25–34 age brackets, constituting 27.6% and 25.8%, respectively. Regarding occupation, the participant pool encompasses a range of professions, with the largest cohort being executive managers/directors at 24.9% (112), trailed by students at 31.6% (142), and teachers/professors at 22.7% (102). Educational attainment spans from primary education to doctoral degrees, with a significant portion holding a bachelor’s degree or equivalent tertiary education level at 34.7% (156), and 32.0% (144) holding doctoral degrees. Proficiency in AI varies, with 56.4% (254) exhibiting basic knowledge, 2.2% (10) indicating no knowledge, and an equal 2.2% (10) possessing advanced knowledge of AI. These demographic particulars present a comprehensive overview of the sample, illustrating the diversity of participants encompassed in this study.

### 3.4. Ethical Considerations

To ensure the appropriate standards of ethical consideration, participants were fully informed about the aims of the study. They were guaranteed confidentiality of their responses, and provided verbal informed consent before the interviews in the qualitative phase and before the surveys in the quantitative phase. All steps of study were approved by the King Faisal University Ethical Committee. The research team identified participants through the researchers’ networks, including colleagues (i.e., professors), students, friends, and family members. They all acknowledged that their participation was voluntarily and data collection is for research purposes.

## 4. Results

### 4.1. The Results of EFA

The results of EFA and reliability analysis using Cronbach’s Alpha coefficient were undertaken to test four distinct constructs: The Need for Personal Contact, Perceived Technological Dependence, General Skepticism towards AI, and Resistance to Use AI. For The Need for Personal Contact construct, the EFA revealed a unidimensional factorial structure with a high Kaiser–Meyer–Olkin (KMO) index of 0.922, indicating the data’s suitability for factorial analysis. The Bartlett’s Test also yielded a significant *p*-value of 0.000, suggesting significant correlations among the items. Moreover, the construct demonstrated excellent internal consistency, with a Cronbach’s Alpha of 0.922, reflecting the reliability of the measurement scale. The EFA explained 94.705% of the variance, highlighting the factor’s substantial explanatory power.

The Perceived Technological Dependence construct exhibited a unidimensional structure, supported by a KMO index of 0.851 and a significant Bartlett’s Test (*p* = 0.000). The Cronbach’s Alpha of 0.970 confirmed strong internal consistency, ensuring the reliability of the measurement scale. The EFA accounted for 91.977% of the variance, indicating the factor’s considerable influence. The General Skepticism towards AI construct also displayed a unidimensional factorial structure, with a KMO index of 0.787 and a significant Bartlett’s Test (*p* = 0.000). The high Cronbach’s Alpha value of 0.976 showed excellent internal consistency and reliability of the scale. The EFA explained 95.370% of the variance, underlining the factor’s substantial contribution to the construct. Finally, the Resistance to Use AI construct exhibited a unidimensional structure, supported by a KMO index of 0.889 and a significant Bartlett’s Test (*p* = 0.000). A Cronbach’s Alpha of 0.980 verified strong internal consistency and reliability. The EFA accounted for 92.495% of the variance, indicating the factor’s meaningful explanatory power.

In conclusion, the EFA and reliability testing results affirm the one-dimensionality and psychometric qualities of the measurement scales for all four constructs. The data’s suitability for factor analysis, high internal consistency, and substantial explanatory power of the factors supports the robustness and reliability of the scales, making them suitable for further research and assessment.

### 4.2. The Results of CFA

We undertake several steps to evaluate the overall measurement model. Initially, we assess the model’s goodness of fit by examining various fit indices such as Chi-squared (Chi2), Goodness of Fit Index (GFI), Adjusted Goodness of Fit Index (AGFI), Root Mean Square Residual (RMR), and Root Mean Square Error of Approximation (RMSEA), among others. Subsequently, we proceed with an examination of the convergent and discriminant validity of the various constructs present in our measurement model.

#### 4.2.1. Goodness-of-Fit Indices for the Measurement Model

Based on the model-fit indicators and following the inclusion of adjustments recommended by the modifications indices such as introducing correlations between errors (e1/e2), (e8/e10), and (e15/18), the model exhibited satisfactory and acceptable indicators of goodness-of-fit. The ratio of Chi-squared to degrees of freedom (χ2/df) equated to 2.936, falling within the suggested range of 1 < χ2/df < 3. The Root Mean Square Error of Approximation (RMSEA) stood at 0.042, a value below the threshold of 0.10, implying a reasonable degree of fit. The Root Mean Square Residual (RMR) amounted to 0.033, which is below the recommended threshold of 0.05. The Comparative Fit Index (CFI) registered at 0.957, indicative of a well-fitting model. The Goodness of Fit Index (GFI) equated to 0.922, and the Adjusted Goodness of Fit Index (AGFI) surpassed 0.8, both signifying an acceptable level of fit. Additionally, the Normed Fit Index (NFI) exceeded the suggested threshold of 0.9, reaching a value of 0.952. Taken together, these outcomes collectively indicate that the modified model aligns effectively with the observed data, presenting a suitable depiction of the underlying constructs.

#### 4.2.2. Convergent and Discriminant Validity

In terms of confirming the convergent validity, we assessed two criteria. It is required that there should be a significant connection between the latent variable and each of its indicators. Through the use of Student’s t-test, we observed that all of the influences of factors are notably significant, with a significance level set at *p* = 0.001. This requirement is duly established. An Average Variance Extracted (AVE) exceeding 0.5 is recommended as per the guidelines by Fornell and Larcker (1981). This suggests that more than 50% of the shared variance between the latent variable and its indicators should be present. When the AVE surpasses this threshold, it signifies that the impact of item variance outweighs the influence of measurement errors. As detailed in the provided table (Table 6), this criterion is satisfactorily met. Furthermore, the provided data indicates strong internal consistency and reliability within the studied constructs. The construct “The Need for Personal Contact” has a high Composite Reliability of 0.989, while “Perceived Technological Dependence” shows 0.974, “General Skepticism Towards AI” scores 0.976, and “Resistance to Use AI” demonstrates an impressive 0.981. These high values emphasize the dependable and consistent nature of measurements for these constructs, bolstering the credibility of this study’s findings (Table 6).

The discriminant validity of the underlying factors can be assessed by showcasing that the extent of shared variance between each variable and its associated items is larger than the shared variance with other variables. For this purpose, we contrasted the relationship between latent variables and the square root of the average variance extracted (AVE). Findings indicate the fulfillment of this criterion, approving discriminant validity.

### 4.3. The Results of SEM

**Figure 3:**
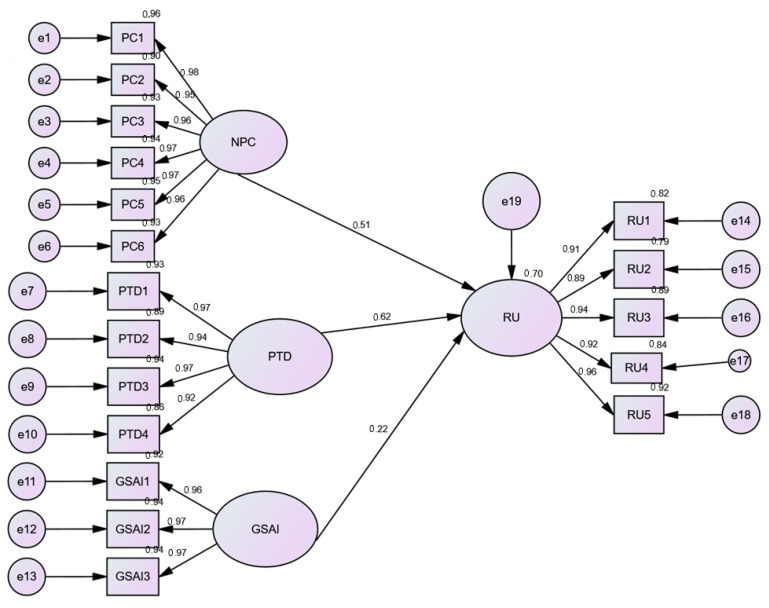
Structural model.

Following the adjustment of the measurement model, a second section is proposed to test the conceptual model of the research. The model testing is conducted through the use of structural equation modeling methods, the steps of which are detailed and explained. The interpretation of the research hypothesis test results is done in two phases: in the first phase, we ensure the quality of fit of the structural model, and in the second phase, we examine the significance and direction of the postulated cause-and-effect relationships. The goodness-of-fit of the structural model was comparable to the previous CFA measurement model. The recorded values are χ²/df = 3.21 (1 < χ²/df < 5), RMSEA: 0.055 (<0.08), RMR: 0.038 (<0.05), CFI: 0.913, GFI: 0.905, NFI: 0.915 (>0.90), (TLI): 0.910 (>0.90), and AGFI: 0.845 (>0.80). These fit indices provide evidence of adequate fit between the hypothesized model and the observed data (Byrne, 2001). Figure 3 depicts the structural model of our study. It illustrates the relationships among the latent variables in the study: NPC (Need for Personal Contact), PTD (Perceived Technological Dependence), GSAI (General Skepticism Toward AI), and RU (Resistance to Use AI).

#### The Links Between Psychological Factors and Resistance to Use AI

The analysis tested the direct links between psychological factors (the Need for Personal Contact, Perceived Technological Dependence, and General Skepticism towards AI) and patients’ resistance to the use of AI in healthcare (See Figure 3 and Table 7). The results strongly supported all three hypotheses, confirming the significant relationships between these factors and resistance to the use of AI. The need for personal contact exhibited a notable and positive connection (β = 0.515, *p* < 0.001), highlighting the impact of seeking interpersonal engagement on consumers’ resistance to adopting AI. Perceived technological dependence displayed a significant relationship (β = 0.620, *p* < 0.001), emphasizing the role of perceptions about reliance on technology in influencing resistance to AI adoption. Finally, general skepticism towards AI showed a marked positive association (β = 0222, *p* < 0.001), underscoring the role of skepticism in shaping consumers’ resistance to use of AI. These findings underscore the pivotal influence of the need for personal contact, perceived technological dependence, and general skepticism toward AI in shaping consumer behaviors concerning AI resistance.

## 5. Discussion

We examined the direct influence of psychological factors: the need for personal contact, perceived technological dependence, general skepticism towards AI, and patients’ resistance to using AI in healthcare. This led to the formulation of three hypotheses: H1: The need for personal contact is positively associated with the Tunisian patient’s resistance to use AI. H2: Perceived technological dependence is positively associated with the Tunisian patient’s resistance to use AI. H3: General skepticism towards AI is positively associated with the Tunisian patient’s resistance to use AI. The analysis of the data supported all three hypotheses, indicating the significant role of these functional and organizational factors in shaping individuals’ resistance to using AI technology.

Our findings revealed that certain individuals exhibit resistance to adopting AI in healthcare, primarily driven by a preference for personal interaction. Patients often perceive that technology cannot adequately replace the human touch and interpersonal engagement crucial in healthcare settings. This observation aligns with earlier research indicating that the acceptance of technology-enabled services hinges on the perceived necessity or preference for personal contact (Walker & Johnson, 2006; Mani & Chouk, 2018). As consumers, patients tend to perceive higher risks, leading them to prioritize direct contact with healthcare professionals to promptly address potential issues. The preference for face-to-face interaction becomes particularly evident when patients need immediate resolution of queries or concerns, or when they wish to express grievances. Many individuals consistently prioritize personal connection and engagement regardless of the situation, valuing the emotional support and rapport established with their healthcare providers, which they fear AI technology may not sufficiently replicate.

Moreover, according to our findings, perceived technological dependence is another factor that may influence patients’ resistance to adopting AI in healthcare. These findings are consistent with the work of Refs. (Licoppe & Heurtin, 2001; Park et al., 2013). Some patients may feel uneasy about relying on technology for their medical care, fearing that it could make them overly dependent on it. They may worry about the consequences of a system failure or malfunction, or they may feel that relying on technology undermines the role of human interaction and decision-making in healthcare. Mani and Chouk (2017) found that automated machines could produce psychological and functional dependence, which may lead to a resistance reaction. For some patients, the idea of being dependent on technology for their healthcare needs can feel unsettling. They value the human touch and the reassurance that comes with knowing that a healthcare provider is involved in their care. This perceived dependence on technology can lead to resistance to adopting AI in healthcare.

Finally, the findings highlight the central role of skepticism in explaining consumer resistance. This finding aligns with previous research, which has pointed out that consumers can display their doubt for both the communication and assertions made about intelligent devices. This skepticism can potentially lead to consumer resistance, as observed in studies by Morel and Pruyn (2003) and Mani and Chouk (2018). Specifically, AI technology generates greater skepticism because they are new results in their first stage of development. Thus, “a resistant buyer usually begins with a high level of skepticism and becomes progressively more accepting with repeated exposure to the product” (Rackham, 1988).

## 6. Implications

This article offers significant contributions to both theory and practice in the field of AI resistance in healthcare. Firstly, it adopts a patient-centric perspective, recognizing the vital role of patients as recipients and beneficiaries of healthcare services. This approach allows for an exploration of psychological factors influencing patients’ resistance to AI technology in healthcare. Understanding patients’ perspectives is crucial for healthcare providers and hospitals aiming to effectively implement AI-based systems, as patient acceptance and engagement are fundamental to their successful integration into healthcare delivery. Secondly, the article employs a mixed-method, merging quantitative analysis and qualitative exploration. This integration of methods offers a comprehensive understanding of the impact of psychological factors on AI resistance from a patient-centric viewpoint. The quantitative analysis offers statistical evidence and insights into the link between factors such as the need for personal contact, perceived technological dependence, general skepticism towards AI, and AI resistance. The qualitative exploration delves deeper into patients’ experiences, perceptions, and attitudes toward AI-based devices, capturing nuanced insights that quantitative analysis alone may not reveal. This mixed-method approach strengthens the validity and richness of the findings, providing a more robust understanding of patients’ resistance to AI technology in healthcare.

From a managerial standpoint, these results offer valuable guidance for healthcare administrators and policymakers tasked with facilitating the successful integration of AI technology. The observed significance of the Need for Personal Contact implies that strategies designed to incorporate AI must also emphasize the preservation of human interactions. Health institutions could consider hybrid approaches that amalgamate AI efficiency with the comfort of personalized care, particularly in situations demanding immediate attention or resolution. Moreover, recognizing the impact of Perceived Technological Dependence calls for proactive measures to address patients’ concerns over technology-driven disruptions. Tailoring communication and education efforts to alleviate these apprehensions could enhance AI acceptance. Furthermore, acknowledging the central role of general skepticism towards AI emphasizes the importance of building trust and familiarity with AI applications. Healthcare administrators should consider gradual and informed exposure to AI technology, as suggested by Rackham (1988), to foster increased acceptance over time. These insights collectively underscore the need for comprehensive change management strategies that take into account the complex connection of psychological factors when introducing AI solutions in healthcare settings. By accounting for these implications, stakeholders can facilitate a smoother transition toward AI integration, ultimately enhancing patient experiences and outcomes.

## 7. Limitation and Further Research Opportunities

While this research has valuable insights, it has some limitations, which could be addressed in future research. Firstly, this research was conducted in a specific healthcare setting, which is the Tunisian healthcare sector, possibly limiting its ability to fully capture the diversity of patient populations. Future studies should replicate the findings in different healthcare contexts and include a more diverse sample of patients. Secondly, this study focused on the direct links between psychological factors and the resistance to the use of AI, without considering other potential mediating or moderating variables related to patients. Hence, future studies could examine the moderating role of patient’s gender, age, and education in these relationships.

## 8. Conclusions

In conclusion, this research significantly contributes to our understanding of the factors influencing patients’ resistance to adopting AI technology in healthcare. The objective of this study was to determine the key factors driving patient resistance to AI adoption. Given the multitude of resistance-related factors, the research employed a two-phase approach. Initially, a qualitative study was conducted to discern the most adaptable factors within the healthcare context. Subsequently, a quantitative study was undertaken to statistically validate the results of the qualitative findings. The strong empirical support for the formulated hypotheses underscores the crucial role of psychological factors, including the need for personal contact, perceived technological dependence, and general skepticism towards AI, in shaping individuals’ attitudes toward AI integration. The findings emphasize the complexity of human psychology in the context of technological innovation, and offer insights that have both theoretical and practical implications. Healthcare stakeholders can benefit from these insights by designing strategies that balance AI efficiency with the human touch, addressing concerns related to technological dependence, and gradually building trust to mitigate skepticism. While this research contributes valuable insights, this study has limitations. Firstly, this research was conducted in a specific healthcare setting, which is the Tunisian healthcare sector, possibly limiting its ability to fully capture the diversity of patient populations. Future studies should replicate the findings in different healthcare contexts and include a more diverse sample of patients. Secondly, this study focused on the direct links between psychological factors and the resistance to the use of AI, without considering other potential mediating or moderating variables.

## Figures and Tables

**Table 1:** Overview of AI applications in healthcare (source: Olawade et al., 2023).

AI Techniques	Application in Healthcare
Medical Imaging Analysis (Oren et al., 2020; Manco et al., 2021; Pinto-Coelho, 2023).	Enhanced Precision and Efficiency: AI significantly boosts the efficiency of medical imaging analysis through automated detection and interpretation. Critical for Diagnoses and Treatment: This technology is vital for detecting and handling conditions such as heart disease and eye disorders. Deep Learning Algorithms: Utilizes neural networks to analyze large datasets, representing a particularly promising application in medical imaging.
Predictive Analytics (Ghaffar Nia et al., 2023).	Improves Patient Outcomes and Reduces Costs: AI-driven predictive analytics enhances patient care and lowers expenses by pinpointing individuals at threat of diseases and offering tailored interferences. Data Analysis: Analyzes data from electronic health records (EHRs). Risk Stratification: Recognizes high-risk individuals for targeted interventions, a crucial application of AI in healthcare.
Charting, Chatbots, and Virtual Assistants (Agatstein, 2023; Alowais et al., 2023).	Automatic Chart Note Generation: AI-enabled charting solutions automatically generate chart notes by analyzing patient data. Enhanced EHR Accuracy and Completeness: Improves the accuracy and completeness of electronic health records (EHRs). Time Savings: Saves time for healthcare professionals by automating data entry. Data Extraction: Extracts information from both structured data (e.g., lab results) and unstructured data (e.g., free-text notes).
AI-driven Robots (Deo & Anjankar, 2023; Denecke & Baudoin, 2022).	Automation of Repetitive Processes: AI robots automate repetitive tasks, enhancing efficiency. Targeted Interventions: Deliver personalized interventions, improving patient results and saving expenses. Types of AI Robots: Includes exoskeletons, mobile, and humanoid robots. Rehabilitation Applications: Primarily used in rehabilitation to aid physical therapy exercises, improving functional and motor capabilities. Geriatric Care: Assist with daily activities and provide companionship in geriatric care.
Virtual Screening (Carracedo-Reboredo et al., 2021)	Virtual Screening: AI-based algorithms used to examine large databases. Database Types: Includes scientific publications, clinical trials, and chemical databases. Identification of Drug Targets: Identifies potential drug targets. Prediction of Efficacy and Safety: Predicts the efficacy and safety of new compounds. Accelerated Drug Discovery: Speeds up the drug discovery process.

**Table 2:** Innovation resistance theories.

Theory	Description	Authors
Status Quo Bias	The Status Quo Bias theory provides valuable insights into individuals’ inclination to maintain the status quo rather than embracing new systems, rooted in established psychological principles. It delineates psychological commitment, cognitive misperception, and rational decision-making as pivotal factors influencing decision inertia. While acknowledging the facilitating role of perceived value, the theory may oversimplify intricate decision processes and neglect external factors impacting decision-making dynamics. Despite offering a framework for comprehending resistance to change, it may not comprehensively encapsulate the nuanced dynamics of individual decision-making or accommodate situational influences.	Samuelson and Zeckhauser (1988), H. W. Kim and Kankanhalli (2009), K. Lee and Joshi (2017), Hajiheydari et al. (2021)
Ram (1987) Theory	Proposes two elements influencing resistance to innovation: innovation characteristics and consumer characteristics. Innovation characteristics include the features and effects of new goods on consumers, while consumer characteristics are psychological traits influencing resistance. Ram and Sheth (1989) considered two categories of hurdles to innovation adoption: functional and psychological. The functional ones include subcategories such as usage, value, and risk hurdles, and are active forms of resistance stemming from the innovation’s characteristics and features (Heidenreich & Kraemer, 2016). These hurdles arise when adopting innovation necessitates significant changes, leading to concerns about risk, usage, and value. In contrast, psychological hurdles include traditional and image barriers, rooted in consumers’ existing worldviews and preexisting perceptions and traditions (Yu & Chantatub, 2016).	Ram (1987), Ram and Sheth (1989)
Expanded Ram Model	Researchers expanded the Ram and Sheth model. Laukkanen and Kiviniemi (2010) explored the impact of company information on resistance barriers. Joachim et al. (2018) proposed a broader framework, including a more inclusive classification of product- and service-specific hurdles. Mani and Chouk (2018) introduced additional obstacles: technological vulnerability, and ideological and personal barriers.	Laukkanen and Kiviniemi (2010), Joachim et al. (2018), Mani and Chouk (2018)
Yu and Lee Model	Refined Ram’s model of innovation resistance distinguishes between innovation resistance and hurdles. Yu and Lee proposed that only the customer and innovation aspects in Ram’s model give rise to innovation resistance, while the process of propagation acts as a societal barrier to innovation diffusion (C. Lee & Yu, 1994).	C. Lee and Yu (1994)

**Table 3:** Summary of key recent research on AI resistance in healthcare.

Study	Focus	Key Findings
Alhashmi et al. (2019)	AI adoption barriers in Australian organizations	Identified barriers using the TOE framework; provided insights and a research agenda for executives and managers.
Zhang et al. (2024)	AI in medical education	Review identified challenges including performance improvement, effectiveness, AI training data, and algorithms.
Strohm et al. (2020)	Implementation barriers in clinical radiology	Inconsistent technical performance, unstructured processes, uncertain added value, and varying acceptance/trust.
Cadario et al. (2021)	Resistance to medical AI	Challenges in understanding algorithms and illusory understanding of human decision-making; proposed interventions.
Gao et al. (2020)	Social media analysis of attitudes toward AI doctors	Revealed positive attitudes tempered by concerns about technology maturity and company trustworthiness.
Fujimori et al. (2022)	AI-based decision support systems in emergency departments	Highlighted system performance and compatibility as significant challenges.
Ahmed et al. (2023)	Barriers to AI adoption in healthcare	A systematic review identified hurdles in six key areas: ethics, liability, regulatory, workforce, social, and patient safety; emphasized the need for understanding and overcoming these barriers for effective AI implementation in healthcare.
Bhattacherjee and Hikmet (2007)	Theoretical model of physician resistance to HIT usage	Identified perceived threat and compatibility as key factors in resistance intentions.
Gaczek et al. (2023)	Consumer resistance to AI healthcare recommendations	Impact of diagnosis trustworthiness and health anxiety; social proof as a mitigating factor.
Mugabe (2021)	AI adoption in radiation oncology in New Zealand	Noted low levels of expertise as a hindrance to AI use.
Jussupow et al. (2022)	Professional identity threats in medical AI resistance	Examined perceived self-threat, temporal distance of AI, and differences between students and professionals.
Chaibi and Zaiem (2022)	Barriers to AI adoption among physicians in Tunisia	Poor infrastructure, including financial resources, specialized training, performance risks, perceived costs, technology dependency, and fears of AI replacing human jobs.

**Table 4:** Synthesis of Verbatim.

Theme	Subtheme	Citation Times	% of the Theme	Verbatim	General Explanation
Psychological Factors Affecting Patient Resistance to AI	Need for Personal Contact	38	88.37%	- *“I value the personal connection I have with my doctor. If AI technology takes over, I feel like I won’t have that same level of trust and comfort with my healthcare provider”.* - *“Sometimes I need someone to listen to me and understand what I’m going through emotionally. I don’t think AI technology can provide that kind of support”.* - *“I want my treatment plan to be tailored to my specific needs and preferences. I don’t think AI technology can do that as well as a human doctor”.* - *“I prefer the doctor and not AI because he takes into account my psychological situation and he tries to comfort me if I’m anxious however robots do not have feelings. When I’m ill, I need the doctor to discuss it with me to comfort me. Communication with the doctor makes me feel better and I forget completely about the disease. Sometimes the illness is not related to a specific part of the body but it’s a psychological matter”.* - *“I want the presence of a doctor to communicate with him. I love the interaction between me and my doctors, it makes me feel secure”.* - *“I want to have a personal connection with my healthcare provider and feel like they truly care about my well-being”.* - *“I worry that adopting AI in healthcare encourages a loss of human touch and a more impersonal approach to medicine. That’s not something I want for myself or my family”.* - *“I prefer to see my doctor face-to-face and have a personal connection with them. AI may be efficient, but it lacks the human touch”.* - *“I am hesitant to use AI because I want a doctor who can understand my unique situation and provide personalized care”.* - *“I worry that AI may overlook important details about my health or miss something that a doctor could catch in person”.* - *“I value the trust I have with my doctor, and I’m not sure I’m ready to trust a machine with my health decisions”.*	The sub-theme “Need for Personal Contact” highlights participants’ strong preference for human interaction, empathy, and trust in healthcare settings. Patients express concerns about AI replacing essential human qualities, such as emotional understanding, personalized care, and the human touch. The quotes emphasize that trust, emotional security, and face-to-face communication are vital for their well-being, which they believe AI cannot replicate. Participants also worry about AI’s inability to fully capture nuanced health details that doctors might identify in person.
	Perceived Technological dependence	13	30.23%	- *“I think it’s important to have human interaction and decision-making in healthcare. I don’t like to be too dependent on technology and lose that personal touch”.* - *“I would worry about what would happen if the technology failed or made a mistake. I would feel more comfortable knowing that a human is still involved in my care”.* - *“I have fear that I can’t make decisions about my own health without relying on a machine. I want to be in control of my own care”.* - *“I worry that if I rely too heavily on AI technology, I might start to ignore my own intuition and gut feelings about my health”.*	The sub-theme “Perceived Technological Dependence” reflects participants’ concerns about over-reliance on AI in healthcare. The quotes highlight fears about losing human involvement in decision-making, the risk of technology failures, and the erosion of patients’ personal control over their care. Participants emphasize the importance of maintaining human intuition, judgment, and autonomy, which they feel are diminished when healthcare depends too heavily on AI.
	General Skepticism	17	39.53%.	- *“I’m not sure I trust a machine to tell me what’s wrong with me, I’d rather see a human doctor”.* - *“AI may work well for some things, but healthcare is too complex and personal for a machine to handle”.* - *“I worry that relying on AI could lead to errors or missed diagnoses that a human doctor would catch”.* - *“I just don’t feel comfortable with the idea of a machine making decisions about my health”.* - *“I think there are too many unknowns with AI in healthcare, and I don’t want to be a guinea pig for the technology”.*	The sub-theme “General Skepticism” captures participants’ doubts and lack of confidence in AI technology for healthcare. The quotes reflect concerns about the complexity and personal nature of healthcare, where participants prefer human judgment over machines. Fears of errors, misdiagnoses, and the unknown risks associated with AI highlight a general discomfort and distrust in relying on technology for critical health decisions. Participants also expressed reluctance to adopt AI due to feeling like test subjects for unproven technologies.

**Table 5:** Measurement scales.

Constructs	Measurement Items
The Need for Personal Contact (NPC)	NPC1. “I prefer to deal face-to-face with my doctor”. NPC2. “I am more reassured by dealing face-to-face with my doctor”. NPC3. “My particular service requirements are better served by doctors”. NPC4. “I prefer face-to-face contact to explain what I want to my doctor and to answer my questions”. NPC5. “I feel like I’m more in control when dealing with my doctor than with automated systems”. NPC6. “I like interacting with my doctor and medical staff in general”.
Perceived Technological Dependence (PTD)	PTD1. “I am afraid of becoming dependent on AI technology”. PTD2. “I am afraid that my doctor become dependent on AI technology”. PTD3. “AI technology will reduce my autonomy and my doctor’s autonomy”. PTD4. “I think my social life will suffer from my use of AI technology”.
General Skepticism Toward AI (GSAI)	GSAI1. “I am skeptical about AI technology”. GSAI2. “I do not think AI technology will be successful”. GSAI3. “I doubt that AI technology can actually do what its manufacturers promise”.
Resistance to Use AI (RU)	RU1. “In sum, the possible use of AI technology to manage my health would cause problems that I don’t need”. RU2. “AI technology to manage my health would be connected with too many uncertainties”. RU3. “Using AI technology for managing my health is not for me”. RU4. “I am likely to be opposed to the use of AI technology for managing my health”. RU5. “I do not need AI technology to manage my health”.

**Table 6:** Reliability and validity testing for the constructs of the global measurement model.

	Factor Loadings	CR	AVE	*p*-Value
The Need for Personal Contact		0.989	0.935	
NPC1	0.980			***
NPC2	0.946			***
NPC3	0.966			***
NPC4	0.970			***
NPC5	0.974			***
NPC6	0.966			
Perceived Technological Dependence		0.974	0.902	
PTD1	0.965			***
PTD2	0.944			***
PTD3	0.968			***
PTD4	0.923			
General Skepticism Towards AI		0.976	0.931	
GSAI1	0.959			***
GSAI 2	0.968			***
GSAI 3	0.967			***
Resistance to Use AI		0.981	0.911	
RU1	0.945			***
RU2	0.933			***
RU3	0.966			***
RU4	0.952			***
RU5	0.975			***

Note: *** = *p*-value < 0.001.

**Table 7:** Results of structural equation model analysis.

Hypotheses	Path Coefficient	Standard Error	C.R.	*p* Values	Results
NPC → RU	0.515	0.021	17.058	***	Accepted
PTD → RU	0.620	0.023	18.973	***	Accepted
GSAI → RU	0.222	0.019	7.900	***	Accepted

*** = *p* < 0.001.

## Data Availability

Data are available upon request from researchers who meet the eligibility criteria. Kindly contact the first author privately through e-mail.

## List of Acronym

**Acronym**	**Full Form**
AI	Artificial Intelligence
IoMT	Internet of Medical Things
SEM	Structural Equation Modeling
AMOS	Analysis of Moment Structures
EFA	Exploratory Factor Analysis
CFA	Confirmatory Factor Analysis
SPSS	Statistical Package for the Social Sciences
NVIVO	Qualitative Data Analysis Software
NPC	Need for Personal Contact
PTD	Perceived Technological Dependence
GSAI	General Skepticism toward AI
AIH	AI in Healthcare
SaMD	Software as a Medical Device
TAM	Technology Acceptance Model
UTAUT	Unified Theory of Acceptance and Use of Technology
IRT	Innovation Resistance Theory
ML	Machine Learning
DL	Deep Learning
NLP	Natural Language Processing
